# Genome Editing to Integrate Seed Size and Abiotic Stress Tolerance Traits in Arabidopsis Reveals a Role for DPA4 and SOD7 in the Regulation of Inflorescence Architecture

**DOI:** 10.3390/ijms20112695

**Published:** 2019-05-31

**Authors:** Siyu Chen, Na Zhang, Qimeng Zhang, Ganghua Zhou, Hainan Tian, Saddam Hussain, Sajjad Ahmed, Tianya Wang, Shucai Wang

**Affiliations:** 1 College of Life Sciences, Linyi University, Linyi 276005, China; 23329915.ok@163.com; 2 Key Laboratory of Molecular Epigenetics of MOE, Institute of Genetics and Cytology, Northeast Normal University, Changchun 130024, China; zhangn906@nenu.edu.cn (N.Z.); m1844313247@163.com (Q.Z.); zhough767@nenu.edu.cn (G.Z.); tianhainan2012@126.com (H.T.); botanistonline@yahoo.com (S.H.); botanist1@yahoo.com (S.A.); wangty309@nenu.edu.cn (T.W.)

**Keywords:** seed size, abiotic stress, AITRs, SOD7, DPA4, shoot branching, Arabidopsis

## Abstract

Both seed size and abiotic stress tolerance are important agronomic traits in crops. In Arabidopsis, two closely related transcription repressors DPA4 (Development-Related PcG Target in the APEX4)/NGAL3 and SOD7 (Suppressor of *da1-1*)/NGAL2 (NGATHA-like protein) function redundantly to regulate seed size, which was increased in the *dpa4 sod7* double mutants. Whereas ABA-induced transcription repressors (AITRs) are involved in the regulation of ABA signaling and abiotic stress tolerance, Arabidopsis *aitr2 aitr5 aitr6* (*aitr256*) triple mutant showed enhanced tolerance to drought and salt. Here we performed CRISPR/Cas9 genome editing to disrupt *DPA4* and *SOD7 in aitr256* mutant, trying to integrate seed size and abiotic stress tolerance traits in Arabidopsis, and also to examine whether *DPA4* and *SOD7* may regulate other aspects of plant growth and development. Indeed, seed size was increased in the *dpa4 sod7 aitr256* quintuple mutants, and enhanced tolerance to drought was observed in the mutants. In addition, we found that shoot branching was affected in the *dpa4 sod7 aitr256* mutants. The mutant plants failed to produce secondary branches, and flowers/siliques were distributed irregularly on the main stems of the plants. Floral organ number and fertility were also affected in the *dpa4 sod7 aitr256* mutant plants. To examine if these phenotypes were dependent on loss-of-function of *AITRs*, *dpa4 sod7* double mutants were generated in Col wild type background, and we found that the *dpa4 sod7* mutant plants showed a phenotype similar to the *dpa4 sod7 aitr256* quintuple mutants. Taken together, our results indicate that the integration of seed size and abiotic stress tolerance traits by CRISPR/Cas9 editing was successful, and our results also revealed a role of DPA4 and SOD7 in the regulation of inflorescence architecture in Arabidopsis.

## 1. Introduction

Seed size is one of the most important agronomic traits in plants. Several different types of seed size regulators have been identified. For example, the ubiquitin receptor DA1 [1], the E3 ubiquitin ligases DA2 and EOD1 (Enhancer of *da1-1*)/BB (BIG BROTHER) [2], the ubiquitin-specific protease UBP15 [3], and the CYP78A cytochrome P450 monooxygenase KLUH and EOD3 (Enhancer3 of *da1-1*) [4,5,6], have been reported to regulate seed size in Arabidopsis.

Most of the seed size regulators identified so far in Arabidopsis are transcription factors, including the AP2 (APETALA2)/EREBP (Ethylene Responsive Element Binding Protein) family protein AP2 [7,8,9], the WRKY transcription factors TTG2 (Transparent Testa GLABRA2), MINI3 (MINISEED3) and SHB1 (Short Hypocotyl Under Blue1) [10,11,12,13,14], the auxin response factor ARF2 [15], the bHLH transcription factor ABI5 (ABA Insentive5) [16], the plant-specific VQ motif proteins IKU1 (HAIKU1) and IKU2 [17,18], and the B3 domain transcription factors DPA4 (Development-Related PcG Target in the APEX4)/NGAL3 and SOD7 (Suppressor of *da1-1*)/NGAL2 (NGATHA-like protein) [19,20].

Among these transcription factors, DPA4 and SOD7 have redundant functions in regulating seed size, as the seed size was increased in the *dpa4 sod7* double mutant [20]. Cotyledon and flower size was also affected in the *dpa4 sod7* double mutant [20], but it is unclear whether DPA4 and SOD7 may regulate other aspects of plant growth and development at mature stages.

Abiotic stress tolerance is also one of the most important agronomic traits in plants. ABA (abscisic acid) is the key plant hormone that regulates plant responses to abiotic stresses including drought, cold, heat and salt [21,22,23]. ABA signaling is controlled by several different regulators including the PYR/PYL/RCAR receptors, the phosphatases PP2Cs (A-group Protein Phosphatase 2C), the protein kinases SnRKs (Nonfermenting 1 (SNF1)-Related Protein Kinases), the ABF/AREB/ABI5-type bZIP transcription factors, and the E3 ligases such as KEG (Keep on Going), DWA1 and DWA2 [24,25,26,27,28,29,30,31,32]. Changes in the expression level of these ABA signaling regulator genes affect ABA signaling, resulting in alteration of plant tolerance to abiotic stresses. For example, overexpression of *PYLs* improved drought tolerance [33,34], whereas loss-of-function of *SnRKs* or ABF/AREB/ABI5-type bZIP transcription factor genes reduced drought tolerance [35,36].

Previously we have identified AITRs (ABA-induced transcription repressors) as a novel transcription factor family [23]. We found that AITRs function as an additional feedback regulatory loop in ABA signaling, and thereby is involved in the regulation of plant response to abiotic stresses [23]. The loss-of-function mutant *aitr2 aitr5 aitr6* (*aitr256*) showed dramatically enhanced tolerance to drought and salt [23].

The CRISPR RNA-guided Cas9 endonuclease is able to cleave double-stranded DNA targets complementary to the guide RNA, and can be used for genome editing in eukaryotic cells to generate mutations at the target sites [37,38]. So far the CRISPR/Cas9 techniques have been successfully used for genome-editing to generate transgene-free targeted mutations in different plant species including the model plant Arabidopsis and crops such as rice [39,40,41,42]. Due to its transgene-free nature, CRISPR/Cas9 genome-edited plants may have a bright future for commercial applications in plant breeding.

Considering that DPA4 and SOD7 negatively regulate seed size [20], while AITRs negatively regulate abiotic stress tolerance [23], we performed genome editing for *DPA4* and *SOD7 in aitr256* mutant, trying to integrate different important agronomic traits in plants by using CRISPR/Cas9 genome-editing. We found that the *dpa4 sod7 aitr256* quintuple mutants obtained produced larger sized seeds, and showed enhanced tolerance to drought. In addition, we also found that DPA4 and SOD7 play a role in regulating shoot branching, floral organ development and fertility in Arabidopsis.

## 2. Results

### 2.1. Seed Size is Increased in the dpa4 sod7 aitr256 Quintuple Mutant Plants

It has been shown that DPA4 and SOD7 function redundantly to regulate seed size in Arabidopsis, as the *dpa4 sod7* double mutant produced larger seeds [20]. Our previously experiments showed that AITRs are a novel transcription factor family that is involved in the regulation of ABA signaling and plant response to abiotic stress tolerance, as the *aitr256* triple mutants showed enhanced tolerance to ABA and abiotic stresses including drought and salt [23]. Considering that both seed size and abiotic stress tolerance are most important agronomic traits in plants, we wanted to examine whether it is possible to integrate the larger seeds and enhanced abiotic stress tolerance traits caused by loss-of-function of *AITRs*, *DPA4* and *SOD7* by generating *dpa4 sod7 aitr256* quintuple mutants.

**Figure 1:**
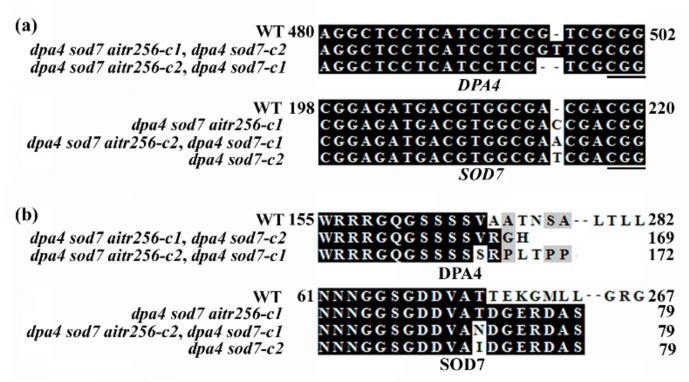
Generation of *dpa4 sod7 aitr256* quintuple and *dpa4 sod7* double mutants. (**a**) Alignment of CRISPR/Cas9 targeted nucleotide sequences of *DPA4* and *SOD7* in Col wild type and the *dpa4 sod7 aitr256* quintuple and *dpa4 sod7* double mutants. The mutants were obtained by transforming *aitr256* triple mutant and Col wild type plants, respectively with *DPA4* and *SOD7* targeting *pHEE* construct, sequencing to examine the editing status in T1 generation, and selecting *Cas9*-free homozygous mutants in T2 and/or T3 generations. Nucleotide numbers are relative to the start codon. Underlines indicate the PAM sites. (**b**) Alignment of the amino acid sequences of DPA4 and SOD7 in the Col wild type and *dpa4 sod7 aitr256* quintuple and *dpa4 sod7* double mutants. Coding sequences of *DPA4* and *SOD7* were subjected to ORF analysis by using ORFfinder (https://www.ncbi.nlm.nih.gov/orffinder/), and predicted amino acid sequences were used for alignment with the amino acid sequences of DPA4 and SOD7, respectively. Identical amino acids are shaded in black and similar in gray. Amino acid numbers are relative to the Met encoded by the start codon.

CRISPR/Cas9 Genome editing is able to generate transgene-free mutant plants [39,40,41,42], we thus used CRISPR/Cas9 to edit *DPA4* and *SOD7 in the aitr256* triple mutants. Two independent *Cas9*-free homozygous mutant lines, i.e., *dpa4 sod7 aitr256-c1* and *dpa4 sod7 aitr256-c2* were generated. In both lines, a single nucleotide insertion or deletion was occurred for *DPA4* and *SOD7* (Figure 1a to a few amino acid substitutions and premature stops for DPA4 and SOD7, respectively (Figure 1b). 

**Figure 2:**
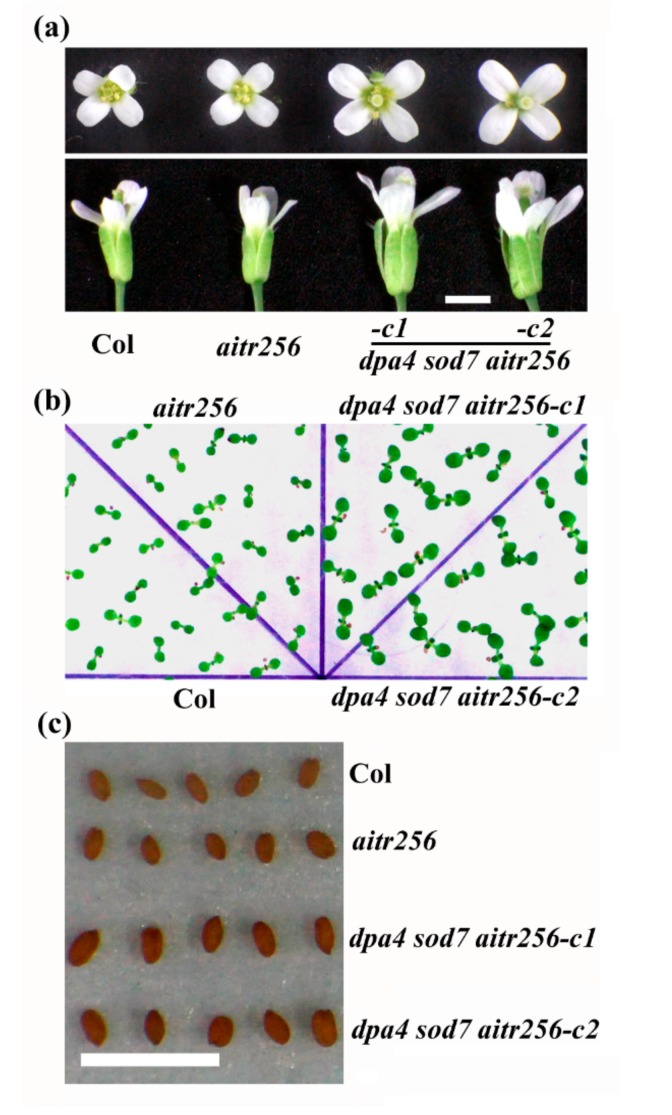
Organ and seed size in the *dpa4 sod7 aitr256* quintuple mutants. (**a**) Flowers of the Col wild type, the *aitr256* triple and the *dpa4 sod7 aitr256* quintuple mutants. Pictures were taken from the third flower on the main inflorescence stem of the plants indicated. (**b**) Seedlings of the Col wild type, the *aitr256* triple and the *dpa4 sod7 aitr256* quintuple mutants. Seeds were sterilized and then plated on 1/2 MS plates. The plates were kept at 4 °C in darkness for 2 days before transferred to a growth room. Picture was taken 5 days after the transfer. (**c**) Seeds of the Col wild type, the *aitr256* triple and the *dpa4 sod7 aitr256* quintuple mutants. Picture of dry seeds was taken for the plants indicated. Bar, 0.2 cm.

When compared with the Col wild type and the *aitr256* triple mutant, the *dpa4 sod7 aitr256* quintuple mutant plants produced larger flowers (Figure 2a), bigger cotyledons (Figure 2b), and larger seeds (Figure 2c), similar to that observed in the *dpa4 sod7* double mutant [20]. On the other hand, no difference was observed between the Col wild type and the *aitr256* triple mutant (Figure 2). 

### 2.2. The dpa4 sod7 aitr256 Quintuple Mutants are Hyposensitive to ABA and Drought

Having shown that the *dpa4 sod7 aitr256* quintuple mutants produced larger seeds, we further examined ABA response and drought tolerance in the *dpa4 sod7 aitr256* quintuple mutants.

**Figure 3:**
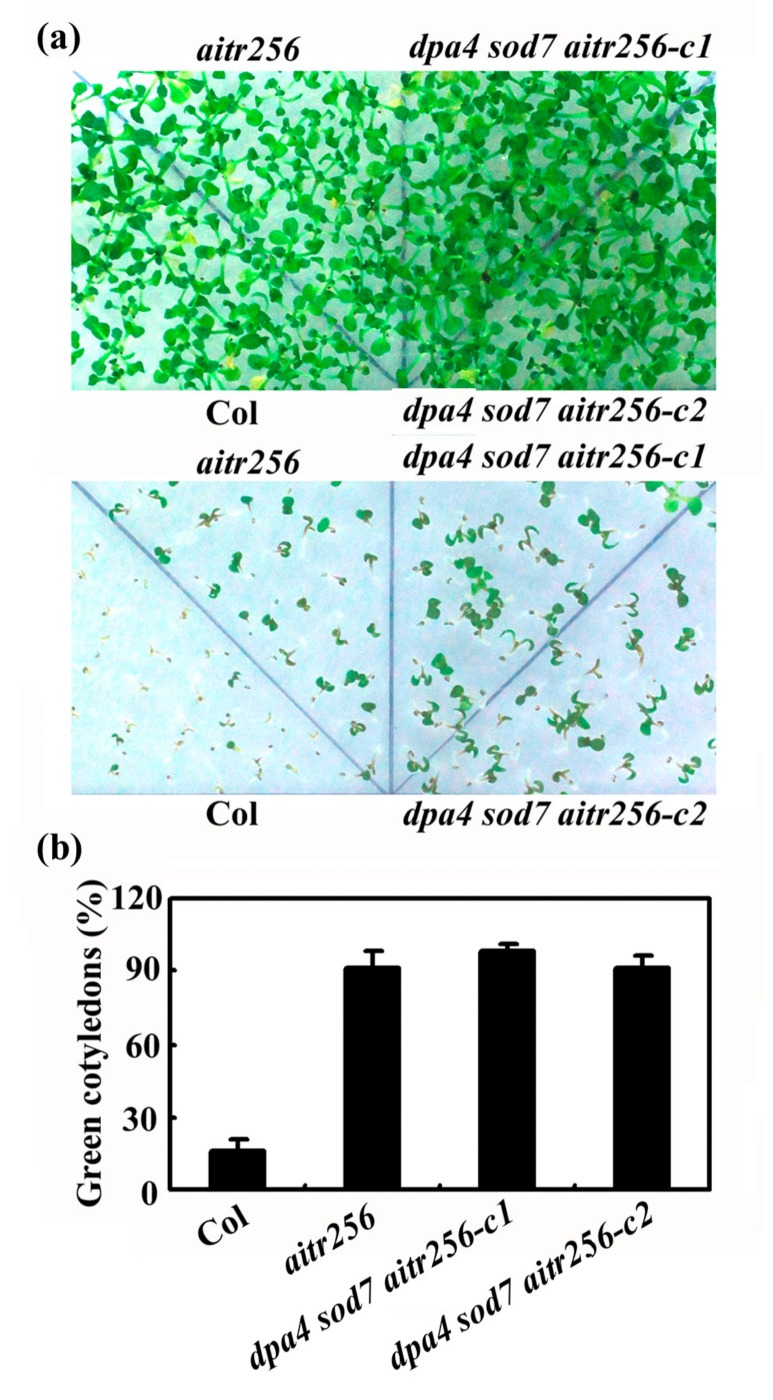
ABA sensitivity of the *dpa4 sod7 aitr256* quintuple mutants. (**a**) ABA sensitivity of the Col wild type, the *aitr256* triple and the *dpa4 sod7 aitr256* quintuple mutants. Seeds were sterilized and then plated on 1/2 MS plates with 2.5 μM ABA, or without ABA as a control. The plates were kept at 4 °C in darkness for 2 days before transferred to a growth room. Pictures were taken 17 days after the transfer. Upper panel, without ABA, lower panel, with ABA. (**b**) Quantitative of green seedlings of the Col wild type, the *aitr256* triple and the *dpa4 sod7 aitr256* quintuple mutants in response to ABA treatment. Seedlings with green cotyledons were counted after the pictures were taken. Data represent the mean ± SD of three replicates.

We first tested ABA response of the *dpa4 sod7 aitr256* quintuple mutants by ABA inhibition of cotyledon greening. To do that, sterilized seeds were germinated and grown on 1/2 MS plates in the presence and absence of 2.5 μM ABA. As shown in Figure 3a, the *dpa4 sod7 aitr256* quintuple mutants showed a reduced ABA sensitivity. Quantitative analyses showed that only ~20% of the Col wild type seedlings produced green cotyledons, whereas that in the *dpa4 sod7 aitr256* quintuple mutants this number raised to ~90% (Figure 3b). No difference in ABA response was observed between the *dpa4 sod7 aitr256* quintuple mutants and the *aitr256* triple mutant (Figure 3).

**Figure 4:**
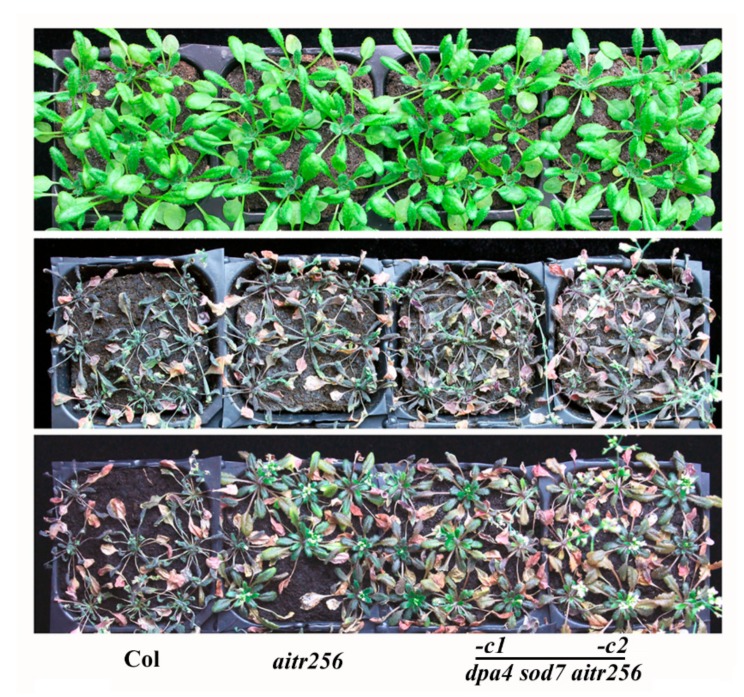
Drought tolerance of the *dpa4 sod7 aitr256* quintuple mutants. Seeds of the Col wild type, the *aitr256* triple and the *dpa4 sod7 aitr256* quintuple mutants were germinated and grown in soil pots for 30 days with sufficient watering. The plants were drought treated by withholding water for 12 days, and then watering was resumed. Pictures were taken before and after drought treatment, and 2 days after watering was resumed. Upper panel, before drought treatment, middle panel, after drought treatment, lower panel, 2 days after water was resumed.

We then examined drought tolerance of the *dpa4 sod7 aitr256* quintuple mutants. The plants were grown in soil for 30 days with sufficient watering, and then were subjected to drought treatment by withholding watering for 12 days. As shown in Figure 4, all the *dpa4 sod7 aitr256* quintuple mutant plants recovered 2 days after re-watering, but all the Col wild type plants failed to recover from the drought treatment (Figure 4). No difference in drought tolerance was observed between the *dpa4 sod7 aitr256* quintuple mutants and the *aitr256* triple mutant (Figure 4). These results indicate that larger size trait in the *dpa4 sod7* double mutant and enhanced abiotic tolerance trait in the *aitr256* triple mutant were successfully integrated by genome editing of *DPA4* and *SOD7* in the *aitr256* triple mutant.

### 2.3. DPA4 and SOD7 Regulate Inflorescence Architecture

**Figure 5:**
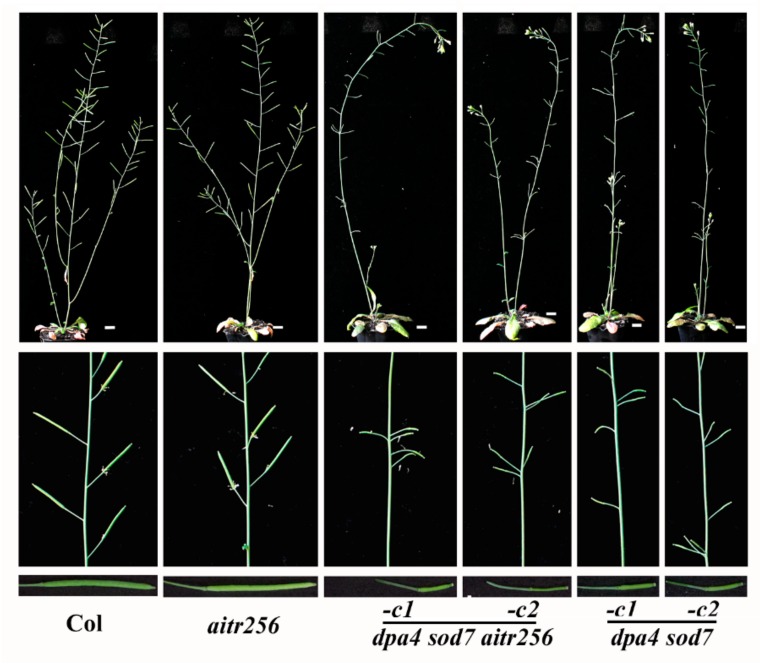
Inflorescence architecture in the *dpa4 sod7 aitr256* quintuple and the *dpa4 sod7* double mutants. Seeds of the Col wild type, the *aitr256* triple, the *dpa4 sod7 aitr256* quintuple and the *dpa4 sod7* double mutants were generated and grown in soil pots for 30 days with sufficient watering. Pictures for inflorescences were taken from 7-week-old plants, for siliques were taken from 6-week-old plants. Upper panel, inflorescences of 7-week-old plants, middle panel, siliques arrangement on the main inflorescence stems, lower panel, 8th siliques on main inflorescence stems.

In addition to the changes previously reported in the *dpa4 sod7* double mutant, such as bigger cotyledons, large-sized flowers and seeds [20], we found that the shoot branching was also affected in the *dpa4 sod7 aitr256* quintuple mutants. In the Col wild type plants, the main inflorescence stem produced a few secondary branches, the siliques distributed almost evenly on the stems, and all except the first 1–2 siliques were able to produce seeds (Figure 5). The *aitr256* triple mutant plants were morphologically indistinguishable to the Col wild type plants (Figure 5). However, in the *dpa4 sod7 aitr256* quintuple mutant plants, the main inflorescence stem failed to produce any secondary branches, clusters of siliques were observed on the main stems, and only a few, especially late generated siliques were able to bear seeds (Figure 5).

**Figure 6:**
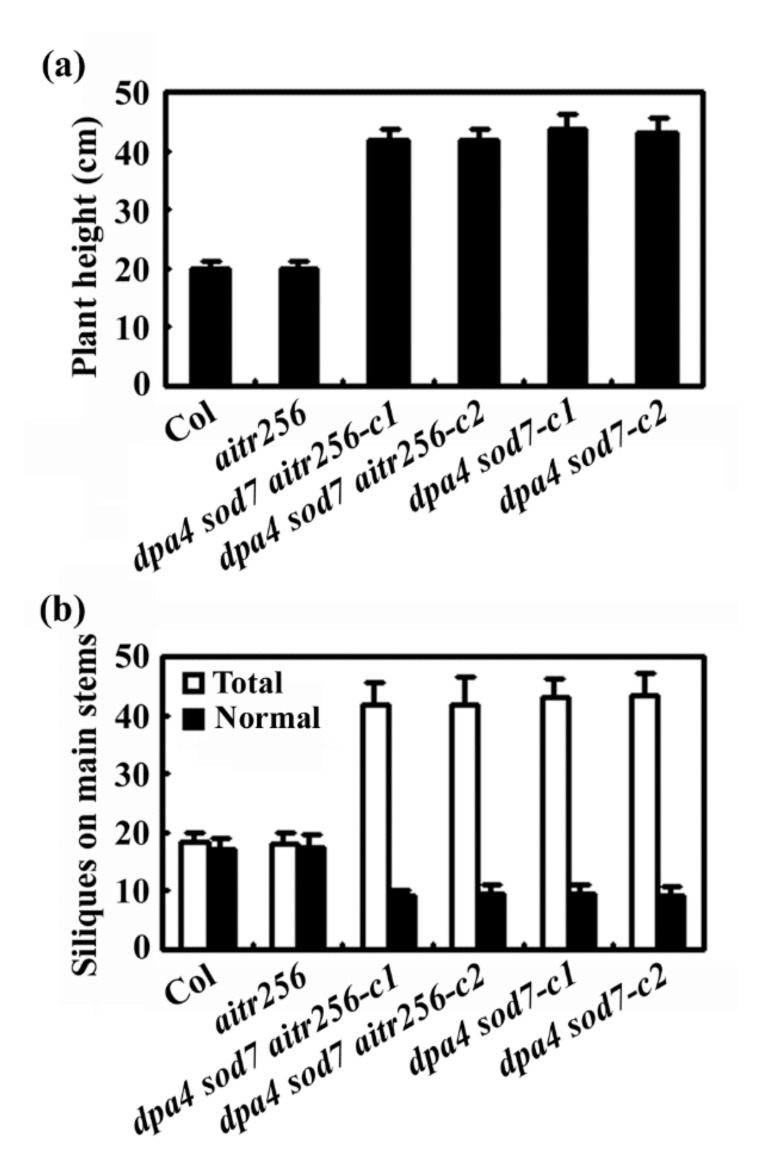
Height and siliques of the *dpa4 sod7 aitr256* quintuple and the *dpa4 sod7* double mutants. (**a**) Height of 12-week-old Col wild type, the *aitr256* triple, the *dpa4 sod7 aitr256* quintuple and *dpa4 sod7* double mutants. (**b**) Siliques of Col wild type, the *aitr256* triple, the *dpa4 sod7 aitr256* quintuple and *dpa4 sod7* double mutants. Seeds of the Col wild type, the *aitr256* triple, the *dpa4 sod7 aitr256* quintuple and the *dpa4 sod7* double mutants were generated and grown in soil pots for 12 weeks, plant height was measured, total siliques and siliques with seeds (normal siliques) on the main inflorescence stems of the plants were counted. Data represent the mean ± SD of at least 11 plants.

We also noticed that main stems of the *dpa4 sod7 aitr256* quintuple mutants were still able to grow and flower even when the Col wild type and the *aitr256* triple mutant finished flowering (Figure 5). Therefore, the main stems of *dpa4 sod7 aitr256* quintuple mutants eventually reached a height about twice of the Col and the *aitr256* triple mutant (Figure 6a), and produced about twice as much siliques, however, only about one fourth of the siliques in the *dpa4 sod7 aitr256* quintuple mutants were able to produce seeds (Figure 6b).

To examine if the phenotypes observed in the *dpa4 sod7 aitr256* quintuple mutant plants were a synergistic effect between the loss-of-function of *AITR* genes and *DPA4* and *SOD7*, we generated *dpa4 sod7* double mutants by using CRISPR/Cas9 to edit *DPA4* and *SOD7* in Col wild type plants. Therefore, two independent *Cas9*-free homozygous mutant lines, i.e., *dpa4 sod7-c1* and *dpa4 sod7-c2* were generated. Similar to the *dpa4 sod7 aitr256* quintuple mutants, a single nucleotide insertion or deletion occurred in *DPA4* and *SOD7* (Figure 1a), resulting in a few amino acid substitutions and premature stops for DPA4 and SOD7, respectively in both lines (Figure 1b). 

Similar to the *dpa4 sod7 aitr256* quintuple mutant plants, we found that the *dpa4 sod7* double mutant plants were unable to produce secondary branches, cluster siliques were found on the main stems, only a few late generated siliques produced seeds, and reached a higher height (Figure 5 and Figure 6).

### 2.4. DPA4 and SOD7 Regulate Floral Organ Development

**Figure 7:**
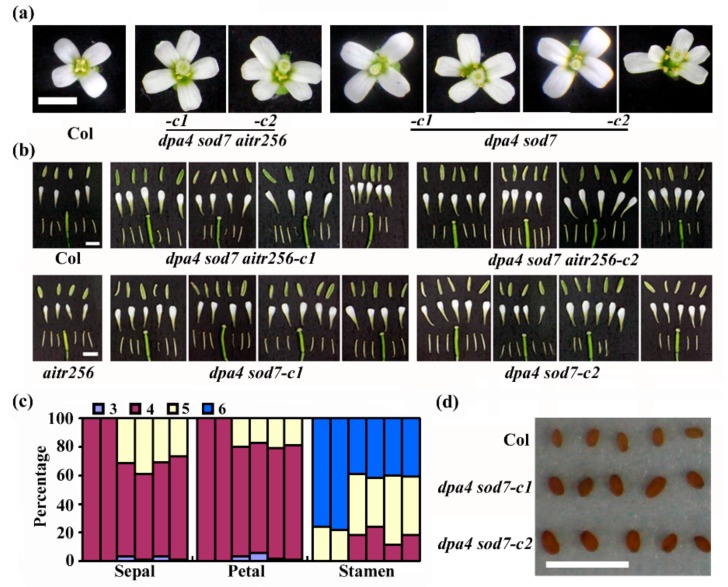
Flower morphology and seed size of the *dpa4 sod7 aitr256* quintuple and the *dpa4 sod7* double mutants. (**a**) Flowers of the Col wild type, the *aitr256* triple, the *dpa4 sod7 aitr256* quintuple and *dpa4 sod7* double mutants. Pictures were taken from flowers on the main inflorescence stems of the plants indicated. (**b**) Floral organs of the Col wild type, the *aitr256* triple, the *dpa4 sod7 aitr256* quintuple and *dpa4 sod7* double mutants, showing irregular number of sepals, petals and/or stamens. (**c**) Percentage of floral organs of the Col wild type, the *aitr256* triple, the *dpa4 sod7 aitr256* quintuple and *dpa4 sod7* double mutants. From left to right, Col, aitr256, *dpa4 sod7 aitr256-c1*, *dpa4 sod7 aitr256-c2*, *dpa4 sod7-c1* and *dpa4 sod7-c2*. A total of 100 flowers for each genotype from the main inflorescence stems of the plants indicated were investigated. (**d**) Seeds of the Col wild type and the *dpa4 sod7* double mutants. Picture of dry seeds was taken for the plants indicated. Bar, 0.2 cm.

In addition to the large-sized flowers, we found that some of the flowers in the *dpa4 sod7 aitr256* quintuple mutant plants have 5 petals (Figure 7a). Furthermore, detailed observation showed that sepal and stamen numbers were also changed in some of the flowers in the *dpa4 sod7 aitr256* quintuple mutant plants (Figure 7b). Quantitative analysis showed that in the Col wild type plants, all the flowers have 4 sepals and 4 petals, about 80% of the flowers have 6 stamens, while the other ~20% have 5 stamens (Figure 7c). In the *dpa4 sod7 aitr256* quintuple mutant plants, the numbers of sepals and petals ranged from 3–5, whereas that of stamens ranged from 4–6 (Figure 7c). Similar changes were observed in the *dpa4 sod7* double mutant plants (Figure 7a–c). As expected, the seed size was increased in the *dpa4 sod7* double mutants (Figure 7d).

## 3. Discussion

CRISPR/Cas9 genome-editing has been successfully used to generate transgene-free mutants in different plant species including Arabidopsis [39,40,41,42]. We provide evidence in this study that CRISPR/Cas9 genome-editing can be used to integrate important agronomic traits in Arabidopsis. In addition, we also found that DPA4 and SOD7, negative regulators of seed size [20], are involved in the regulation of shoot branching and floral organ development.

Both seed size and abiotic stress tolerance are most important agronomic traits in plants. Many reports in Arabidopsis have shown that a lot of transcription factors are involved in the regulation of seed size [8,9,10,11,12,13,14,16,17,18,20,43], as well as abiotic stress tolerance [23,35,36,44]. Among them, DPA4 and SOD7 act redundantly to regulate seed size, as the *dpa4 sod7* double mutant produced larger seeds [20]. AITRs function redundantly to regulate abiotic stress tolerance, as the *aitr256* triple mutant showed enhanced tolerance to abiotic stresses including drought and salt [23]. Generation of *dpa4 sod7 aitr256* quintuple mutants to integrate larger seeds and enhanced abiotic stress tolerance by using traditional means, i.e., crossing, is possible, but identification of homozygous mutants, especially when multiple genes are involved is laborious and protracted.

On the other hand, genome editing by CRISPR/Cas9 has been showed to be an efficient way to generate transgene-free mutants in a much faster manner [39,40,41,42]. Up to eight genes have been successfully edited at the same time by using a single CRISPR/Cas9 construct [39], and CRISPR/Cas9 genome editing has been used to improve important agronomic traits in crops [45]. Therefore, we edited *DPA4* and *SOD7* in the *aitr256* triple mutants to see if we are able to generate mutants that could produce larger seeds and show enhanced abiotic stress tolerance. As expected, we obtained *Cas9*-free *dpa4 sod7 aitr256* quintuple mutants (Figure 1). Similar to *dpa4 sod7* double mutant [20], the *dpa4 sod7 aitr256* quintuple mutants produced larger seeds (Figure 2). Enhanced tolerance to ABA (Figure 3), as well drought (Figure 4), was also observed in the *dpa4 sod7 aitr256* quintuple mutants. These results indicated that the bigger seeds and enhanced abiotic stress tolerance traits were successfully combined in the *dpa4 sod7 aitr256* quintuple mutants, suggesting that CRISPR/Cas9 genome editing can be used to integrate different agronomic traits in plants, which shortens the traditional breeding process significantly.

In addition, we also found that inflorescence architecture was altered in the *dpa4 sod7 aitr256* quintuple mutants, which failed to produce secondary branches, have clustered siliques, and greatly reduced fertility (Figure 5 and Figure 6). The *dpa4 sod7 aitr256* quintuple mutants also produced flowers with altered number of floral organs including sepal, petal and stamen (Figure 7). Since none of these phenotypes had been reported previously, we further generated *dpa4 sod7* double mutants by using CRISPR/Cas9 (Figure 1), to examine if these phenotypes are dependent on *aitr256* mutant background. As expected, the *dpa4 sod7* double mutants produced larger seeds (Figure 7). However, the inflorescence phenotype of the *dpa4 sod7* double mutants was similar to that of the *dpa4 sod7 aitr256* quintuple mutants (Figure 5 and Figure 7). These results suggested that the inflorescence phenotype observed in the *dpa4 sod7 aitr256* quintuple mutants was independent of the *aitr256* mutant background, and DPA4 and SOD7 played a role in the regulation of shoot branching in Arabidopsis.

The *dpa4 sod7 Cas9*-free mutants produced bigger cotyledons, large-sized flowers and seeds, similar to that observed in the *dpa4 sod7* T-DNA insertion mutants [20], indicating that *dpa4 sod7 Cas9*-free mutants are indeed loss-of-function mutants. On the other hand, no morphological changes were observed in the *DPA4* and *SOD7* heterozygous edited T1 plants, and among their offspring, all the phenotypes observed in the *dpa4 sod7* mutants co-segregated with the homozygous edit status of both *DPA4* and *SOD7*, it is very unlikely that the inflorescence phenotype observed in the *dpa4 sod7* mutants were caused by off-target mutation of other genes. Considering that fertility was greatly reduced in the *dpa4 sod7* double mutants (Figure 5), *DPA4* and *SOD7* may not serve as good candidate genes for genome editing by CRISPR/Cas9 to increase yield in plants, even though the *dpa4 sod7* double mutants indeed produced larger seeds (Figure 7).

Shoot branching is another important agronomic traits, which is regulated by the interplay of several different plant hormones including auxin, strigolactone, cytokinins and abscisic acid [46,47,48]. The TCP (TB1/CYCLOIDEA/PCF) transcription factor BRC1 (BRANCHED 1) is the central regulator of shoot branching, which negatively regulates branching in Arabidopsis [49]. All the plant hormones mentioned above and other regulators regulate shoot branching mainly via regulating the expression of *BRC1*, or the activity of BRC1 [47,48]. DPA4 and SOD7 have been shown to function as transcriptional repressors [20], therefore it will be interesting to examine whether DPA4 and SOD7 are also functioning through BRC1 to regulate branching in Arabidopsis.

Specification of floral organs is regulated by several key transcription factors [50], including the MIKCc-type MADS-domain transcription factors AG (AGAMOUS), AP3/PI (PISTILLATA), AP1 [51,52,53,54], and the AP2 [7]. Since floral organ numbers were changed in the *dpa4 sod7* double mutants, it will be also interesting to examine if DPA4 and SOD7 may regulate the expression of the above key transcription factor genes involved in the regulation of floral organ specification.

Nevertheless, our results in the study show that genome editing by CRIPSPR/Cas9 is able to integrate different agronomic traits in plants, and DPA4 and SOD7 play a role in regulating branching and floral organ specification in Arabidopsis.

## 4. Materials and Methods

### 4.1. Plant Materials and Growth Conditions

The Arabidopsis (*Arabidopsis thaliana*) ecotype Columbia-0 (Col) was used as wild type. The *aitr2 aitr5 aitr6* (*aitr256*) triple mutant was in the Col ecotype background, and has been described previously [23].

Arabidopsis seedlings for cotyledon size observation and ABA treatment were grown on 1/2 MS plates. Arabidopsis plants used for plant transformation and phenotypic observation were grown in soil pots. All plants were grown in a growth room with growth conditions described previously [44,55].

### 4.2. Construct

To generate CRISPR/Cas9 construct for genome editing of *DPA4* and *SOD7*, exon sequences of *DPA4* and *SOD7* were subjected to CRISPRscan (http://www.crisprscan.org/?page=sequence) to identify appropriate target sequences. Selected target sequences were then evaluated with Cas-OFFinder (http://www.rgenome.net/cas-offinder/). The target sequences used for editing *DPA7* was 5′-AGGCTCCTCATCCTCCGTCG(CGG)-3′, for *SOD7* was 5′-CGGAGATGACGTGGCGACGA(CGG)-3′. The *pHEE401E* vector was used to generate CRISPR/Cas9 construct by following the procedures described by Wang et al. [56]. The primers used to insert the target sequences into the *pHEE401E* vector were, *DT1-BsF* (*SOD7*), 5′-ATATATGGTCTCGATTGGGAGATGACGTGGCGACGAGTT-3′, *DTI-F0* (*SOD7*), 5′-TGGGAGATGACGTGGCGACGAGTTTTAGAGCTAGAAATAGC-3′, *DT2-R0* (*DPA4*), 5-AACCGACGGAGGATGAGGAGCCCAATCTCTTAGTCGACTCTAC-3′, and *DT2-BsR* (*DPA4*): 5′-ATTATTGGTCTCGAAACCGACGGAGGATGAGGAGCCC-3′. The primers used for colony PCR and to sequence the CRISPR/Cas9 constructs generated were, *U626-IDF*, 5′-TGTCCCAGGATTAGAATGATTAGGC-3′ and *U629-IDR*, 5′-AGCCCTCTTCTTTCGATCCATCAAC-3′.

### 4.3. Plant Transformation, Transgenic plant Selection and Cas9-Free Mutant Isolation

About 5-week-old Col wild type and the *aitr256* triple mutant plants with several mature flowers on the main inflorescence were used for transformation. The plants were transformed with the CRISPR/Cas9 construct generated by using floral dip method [57]. T1 seeds were collected and transgenic plants were selected by plating the seeds on 1/2 MS plates containing 30 μg/mL hygromycin and 50 μg/mL carbenicillin. Gene editing status was examined at T1 generation by amplifying and sequencing the genomic sequence of *DPA4* and *SOD7*. T2 seeds generated from gene edited T1 plants were sown directly into soil pots and used to select homozygous *Cas9*-free mutants. Two independent lines of *Cas9*-free homozygous mutants were obtained and used for the experiments.

### 4.4. DNA Isolation and PCR

To examine gene editing status of *DPA4* and *SOD7*, DNA was isolated from leaves of the transgenic plants, and used as templates to amplify genomic sequences of *DPA4* and *SOD7*, respectively by PCR. PCR products was recovered from gel and used for sequencing. Sequencing results were examined and aligned with wild type genome sequences of *DPA4* and *SOD7*, respectively. To isolate *Cas9*-free mutants, DNA isolated from leaves of the gene edited transgenic plants was used as template to amplify *Cas9* gene fragment by PCR. The primers used for PCR amplification of *DPA4* and *SOD7* were, *DPA4-F*, 5′-ATGTCAGTCAACCATTACTCCAC-3′ and *DPA4-R*, 5′-CATAGTGGGAATACTCTGAGTGAG-3′, and SOD7-F, 5′-ATGTCAGTCAACCATTACCACAAC-3′ and SOD7-R, 5′-AAGAGAACATGTAAATATGCAATACAAATTA-3′. The primers used for PCR amplification of *Cas9* were, *Cas9-F*, 5′-GGACAACGAGGAGAATGAGG-3′, and *Cas9-R*, 5′-TGTCTCGACCAGCTGCCTCTT-3′

### 4.5. ABA Sensitivity Analysis

ABA inhibited cotyledon greening assay was used to examine ABA sensitivity of the *dpa4 sod7 aitr256* quintuple mutants by following the procedure described previously [58,59]. Briefly, sterilized seeds of the Col wild type, the *aitr256* triple and the *dpa4 sod7 aitr256* quintuple mutants were plated on 1/2 MS plates with or without 2.5 μM ABA. The plates were kept at 4 °C in darkness for 2 days, and then transferred to a growth room. Pictures were taken 17 days after the transfer by using a digital camera, seedlings with green cotyledons were counted, and percentage of green cotyledons was calculated. The experiments were repeated three times with similar results.

### 4.6. Drought Tolerance Analysis

Drought tolerance of the *dpa4 sod7 aitr256* quintuple mutants was assayed as described previously [23] with some modifications. In brief, seeds of the Col wild type, the *aitr256* triple and the *dpa4 sod7 aitr256* quintuple mutants were germinated in soil pots and grown for 30 days with sufficient watering. The plants were withheld watering for 12 days, and then watering was resumed. Pictures were taken before and after drought treatment, as well as 2 days after watering was resumed, by using a digital camera.

### 4.7. Morphological Assays

For morphological assays of seedling cotyledons, sterilized seeds of the Col wild type, the *aitr256* triple and the *dpa4 sod7 aitr256* quintuple mutants were plated on 1/2 MS plates. The plates were kept at 4 °C in darkness for 2 days and then transferred to a growth room. Picture of 5-day-old seedlings were taken under a dissection microscope equipped with a digital camera.

For morphological assays of mature plants, seeds of the Col wild type, the *aitr256* triple, the *dpa4 sod7 aitr256* quintuple and the *dpa4 sod7* double mutants were germinated and grown in soil pots. Pictures for the plants and inflorescences were taken at indicated growth stages by using a digital camera. Pictures for flowers and siliques were taken under a dissection microscope equipped with a digital camera. Flowers were dissected under a dissection microscope and pictures were taken by using a digital camera. Twelve-week-old plants that have stopped flowering were used for height measurement and siliques counting.
